# Two‐ and three‐piece implants to boost data generation in preclinical in vivo research—A short technical report

**DOI:** 10.1002/cre2.805

**Published:** 2023-10-31

**Authors:** Andreas Stavropoulos, Benjamin Bellon, Benjamin Pipenger, Ole Z. Andersen

**Affiliations:** ^1^ Department of Periodontology, Faculty of Odontology Malmö University Malmö Sweden; ^2^ Division of Conservative Dentistry and Periodontology, University Clinic of Dentistry Medical University of Vienna Vienna Austria; ^3^ Department of Periodontology, School of Dental Medicine University of Bern Bern Switzerland; ^4^ Department of Periodontology, Faculty of Dentistry University of Zurich Zurich Switzerland; ^5^ Preclinical & Translational Research Institut Straumann AG Basel Switzerland

**Keywords:** biomechanical, histology, implants, preclinical

## Abstract

The purpose of this technical report is to present two novel experimental implant designs to boost data generation in preclinical in vivo research. Specifically, the report describes the rationale and the components of (1) a two‐piece experimental implant suitable for a small animal platform (e.g., the rabbit femur/tibial epiphysis model), consisting of a threaded apical‐ and a coronal cylindrical piece, which is intended for collecting two types of biomechanical data, and (2) a three‐piece experimental implant suitable for a large animal platform (e.g., the mini‐pig mandible model), consisting of an apical “wound chamber”, which allows the collection of histological/histomorphometrical data, and a middle threaded and coronal cylindrical piece, which also allow the collection of two types of biomechanical data. The increased volume of information generated from a single experiment in a small animal platform, using the proposed two‐piece implant design, may assist in a more qualified decision‐making process, on whether it is relevant to proceed to further assessment using a large animal platform. Furthermore, the increased volume of information generated in a single animal experiment either in a small or large animal platform, using the proposed two‐ and three‐piece implants, respectively, likely decreases the number of animals otherwise needed for collecting the same information with standard one‐piece implants and, thus, contributes to the reduction/refinement elements of the 3R principle.

## 1 BACKGROUND

Preclinical in vivo (i.e., animal) models are commonly used in dental implant research, as an analog to the human, and are considered necessary to study the safety, efficacy, and biocompatibility of new implant technologies before clinical testing (Berglundh & Stavropoulos, 2012). This is because solely cell/tissue or organ cultures cannot reflect the biological action or any toxic/deleterious effects, for example, of a third‐generation implant surface technology, in humans; such in vitro cultures cannot accurately replicate the complex in vivo situation in which a plethora of molecular factors, signals, and cells interrelate in a three‐dimensional environment. Moreover, the use of animals is often less burdensome and usually less costly compared with large‐scale clinical odontological studies (Klinge et al., 2021). As an example, to evaluate healing responses or possible side effects of novel implant surfaces, histology is considered the most accurate method of assessment; however, studies in humans, with intentional implant installation aiming at biopsy harvesting, for example (Lazzara et al., 1999), are not common. Furthermore, major regulatory agencies such as the European Medicines Agency (www.ema.europa.eu) and the US Food and Drug Administration (www.fda.org) often require a series of targeted preclinical in vivo assessments, before allowing clinical trials. In this context, the Declaration of Helsinki from 2008 advocates that biomedical research involving humans must be based on results from animal experiments, however, without compromising the welfare of animals used for research (http://www.wma.net/en/30publications/10policies/b3/).

Several small and large animal models are available in preclinical in vivo testing of implants, and the choice of the model should be based on the particular research question and the type of implant technology to be assessed. In general, a preclinical in vivo model is based on resemblances and analogies between the process and system under study in the animal and the human; the more resemblances—in both health and disease—in the animal and the human, the more appropriate is considered the model; and the information/data obtained can more reliably be translated to the human situation. It is widely accepted that being close phylogenetically and/or showing anatomical resemblance indicates identical molecular background and biochemical mechanisms, and comparable physiology.

Most animal platforms, perhaps except for nonhuman primate models, do not completely reconstruct the anatomical, physiological, biomechanical, and functional settings of the human mouth and jaws. Deviations among species regard aspects of macro‐ and microanatomy and the dimensions of jaws, alveolar processes and teeth, occlusion, amount and character of the gingiva and mucosa, healing rate, and animal behavior (Stavropoulos & Sculean et al., 2015). On the other hand, the more basic the feature, function, or response studied, the more true the comparison between animals and humans. Fundamental biological mechanisms are usually shared among species, including species rather distant from humans in terms of taxonomy. Bone remodeling and bone healing, and thus osseointegration, represent such fundamental biological mechanisms across vertebrates (Pilawski et al., 2021).

In this context, small animal platforms have several advantages over the use of skeletally large animals. The fact they are small in size bypasses issues regarding the housing requirements and husbandry, related to skeletally larger animals. This permits the inclusion of bigger numbers of animals, which permits the collection of enough volume of data for appropriate assessment, with less costs. Handling of small animals is easier than that of large animals. Preparation for and recovery from surgical interventions often is faster, while they can endure larger/longer surgery, seemingly with much less distress and fewer complications than large animals, which, in turn, lessens the risk of losing experimental units during the duration of the experiment. The drawback of using small animals is that only a limited number of experimental sites is often possible per animal, which, in turn, entails that larger numbers may be needed to adequately compare several groups within the same experiment. Furthermore, due to the well‐defined genetic background, and less variation in terms of biological response, as well as due to the much higher metabolic rate and better/faster healing capacity, results from small animal platforms are less translational compared to results from large animal platforms. In perspective, the results in preclinical in vivo models should in some way translate to the highly variable biological background of humans. Thus, it is common that novel implant surface technologies are first screened in a small animal platform, to assess the potential and relevance for further assessment using a large animal platform.

Common methods for assessing the osseointegration potential of an implant surface are various biomechanical tests and histological/histomorphometrical assessments. The purpose of this technical report is to present two novel experimental implant designs to boost data generation in preclinical in vivo research. Specifically, (1) a two‐piece experimental implant suitable for a small animal platform (e.g., the rabbit femur/tibial epiphysis model), which is intended for collecting two types of biomechanical data, and (2) a three‐piece experimental implant suitable for a large animal platform (e.g., the mini‐pig mandible model), which allows collection of two types of biomechanical data and also histological/histomorphometrical data.

## 2 TWO‐PIECE IMPLANT

**Figure 1:**
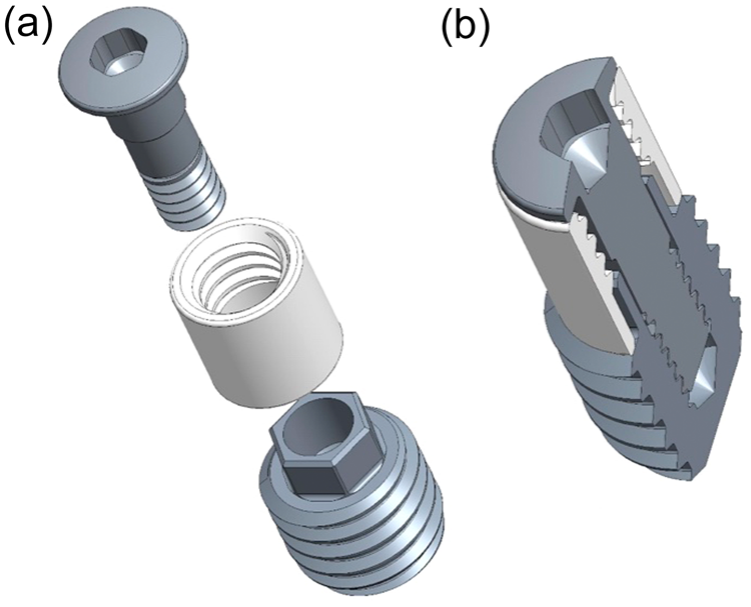
Three‐dimensional reconstruction of the two‐piece implant, including the cover‐screw (a) and in cross‐section when assembled (b).

This implant is parallel‐walled, Ø3.75 mm × 6.5 mm, and comprises two major pieces (Figure 1): (1) a threaded apical one for implant fixation/stabilization and reverse torque testing—this piece also features a 2.2 mm high external‐hex interface at its coronal aspect, enabling connection to the reverse torque device; and (2) a coronal press‐fit cylindrical one for pull‐out testing—this piece features internal threading at its coronal aspect, enabling connection to the tensile (pull‐out) testing device. Additionally, the design includes a cover screw, which seals the internal geometry of the pieces and keeps the two‐piece implant together.

The dimension of the implant allows for the placement of two units (e.g., test and control) in the medial and lateral femoral or tibial epiphyses. The implant socket is gradually enlarged with sequential drilling under continuous physiological saline solution irrigation. The apical (threaded) and the coronal (cylindrical) pieces are assembled and connected with the flat cover screw and inserted in the prepared socket with a hand driver. The implants are installed mono‐cortically and flush to the bone surface, and healing is submerged.

**Figure 2:**
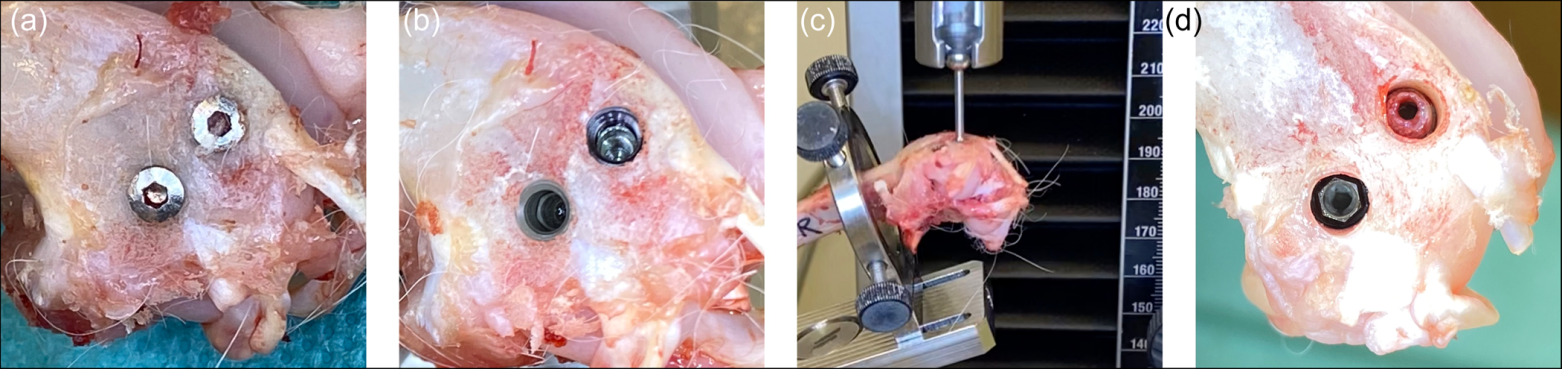
The soft tissues are dissected, the implants exposed (a), and the cover‐screw removed to get access to the internal threading of the coronal press‐fit cylindrical piece of the implant (b), which is then mounted at 90° with a stylus to a tensile‐test machine for pull‐out testing (c). When the pull‐out measurement has been performed, the coronal (cylindrical) piece is removed from the implantation site and the 2.2 mm external hex interface of the apical (threaded) piece of the implant is accessible (d) and can be mounted to a corresponding driver for reverse torque assessment using a torque meter.

Following termination (e.g., after 2–4 weeks of healing), the soft tissues are dissected to expose the implants (Figure 2a) and the cover‐screw is removed to get access to the internal threading of the coronal press‐fit cylindrical piece of the implant (Figure 2b), which is then mounted in 90° with a stylus to a tensile‐test machine for pull‐out testing with a speed of 1.0 mm/min (Figure 2c). When the pull‐out measurement has been performed, the coronal (cylindrical) piece is removed from the implantation site and the 2.2 mm external hex interface of the apical (threaded) piece of the implant is accessible (Figure 2d, left implant), and can be mounted to a corresponding driver for reverse torque assessment using a torque meter.

## 3 THREE‐PIECE IMPLANT

**Figure 3:**
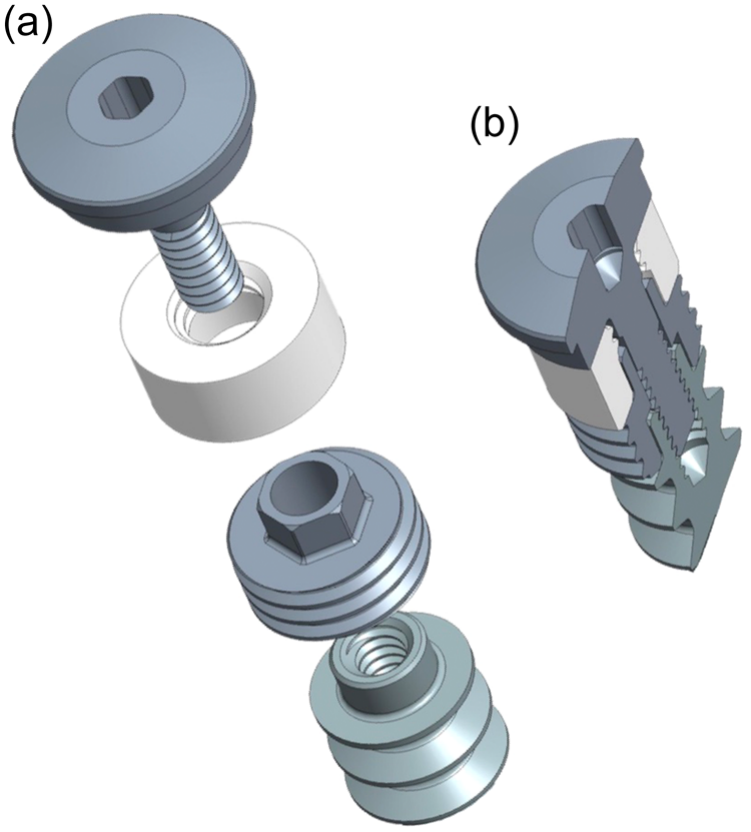
Three‐dimensional reconstruction of the three‐piece implant, including the cover‐screw (a) and in cross‐section when assembled (b).

This implant has a step‐type design, is 9 mm long, and comprises three major pieces: (1) An apical one (Ø4.1 mm) featuring a “wound chamber” for histological assessment—this piece also features a 2.2 cylindrical interface at its coronal aspect with internal threading to accommodate a healing cup; (2) a middle threaded one (Ø4.7 mm) for reverse torque testing—this piece also features a 2.2 mm high external‐hex interface at its coronal aspect, enabling connection to the reverse torque device; and (3) a coronal press‐fit cylindrical one (Ø5.0 mm) for pull‐out testing—this piece features internal threading, enabling connection to the tensile (pull‐out) testing device. Additionally, the design includes a cover screw, which seals the internal geometry of the pieces and keeps the three‐piece implant together (Figure 3).

The dimension of the implant allows for placement of up to six units on each side of the edentulated and healed mandible of the mini‐pig. The implant socket is gradually enlarged with sequential drilling under continuous physiological saline solution irrigation. The apical, middle, and coronal pieces are assembled and connected with the flat healing cap and inserted in the prepared socket with a hand driver. The implants are installed monocortically and flush to the bone surface, and healing is submerged.

**Figure 4:**
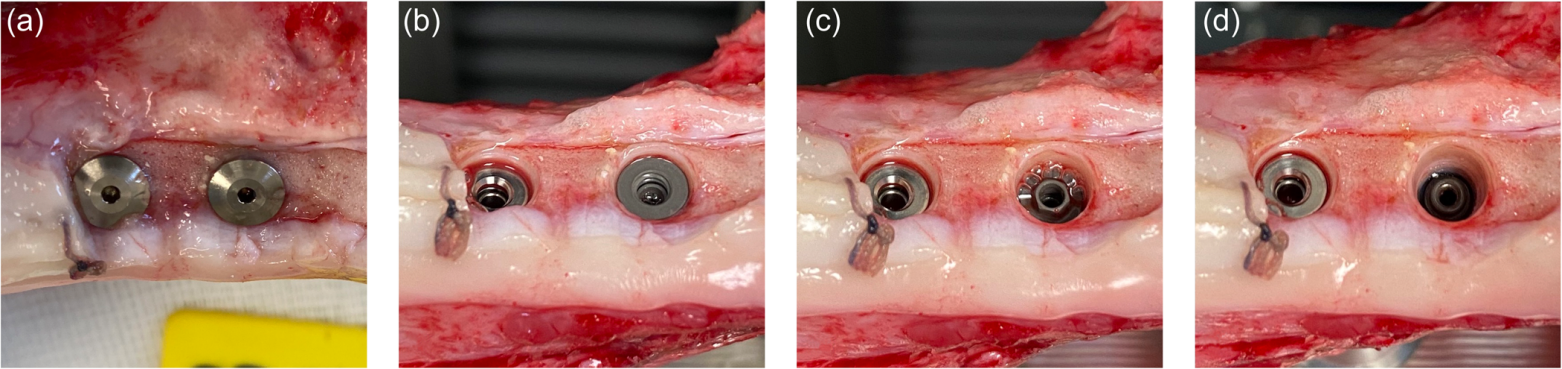
The soft tissues are dissected, the implants exposed (a), and the cover‐screw removed to get access to the internal threading of the coronal press‐fit cylindrical piece of the implant (b), which is then mounted at 90° with a stylus to a tensile‐test machine for pull‐out testing. When the pull‐out measurement has been performed, the coronal (cylindrical) piece is removed from the implantation site and the 2.2 mm external hex interface of the central (threaded) piece is accessible (c; right implant) and can be mounted to a corresponding driver for reverse torque assessment using a torque meter. When the reverse torque measurement has been performed, the central (threaded) piece is removed from the implantation site and the apical (wound chamber) piece of the implant remains in the bone for histology (d; right implant).

Following termination (e.g., after 4 weeks of healing) the soft tissues are dissected to expose the implants (Figure 4a), and the cover‐screw is removed to get access to the internal threading of the coronal press‐fit cylindrical piece of the implant (Figure 4b), which is then mounted in 90° with a stylus to a tensile‐test machine for pull‐out testing with a speed of 1.0 mm/min. When the pull‐out measurement has been performed, the coronal (cylindrical) piece is removed from the implantation site and the 2.2 mm external hex interface of the central (threaded) piece is accessible (Figure 4c; right implant), and can be mounted to a corresponding driver for reverse torque assessment using a torque meter. When the reverse torque measurement has been performed, the central (threaded) piece is removed from the implantation site and the apical (wound chamber) piece of the implant remains in the bone for histology (Figure 4d; right implant).

## 4 DISCUSSION

The two‐ and three‐piece implants presented above boost the volume of data generated in a single animal experiment assessing novel implant surface technologies. In particular, with the two‐piece implant suggested for use in small animal platforms, two types of biomechanical data can be produced. The coronal (cylindrical) piece of the implant contributes with pull‐out force assessment, representing the pure interaction between the implant surface characteristics/chemistry and the bone tissue, while the apical (threaded) piece, contributes with reverse torque force assessment, representing the combined impact of surface characteristics/chemistry and implant macrodesign. As an alternative, for the two‐piece implant, the apical piece can be utilized for histological assessment in combination with the mechanical readout from the coronal cylindrical piece. The three‐piece implant, suggested for use in a large animal platform, has the added feature of a “wound chamber” that allows for histological/histomorphometrical data to be added to the biomechanical data derived from the central and the coronal piece, contributing with reverse torque force and pull‐out force assessment, respectively.

The various biomechanical tests (e.g., pushout, pullout, or reverse torque tests) measure the biomechanical force required to cause implant loosening; this reflects the mechanical properties and strength of the implant‐bone interface and is a relatively valid surrogate measure of osseointegration. Nevertheless, although mechanical performance can indeed be considered as an ultimate test for the success of osseointegration, a comprehensive assessment of a new implant surface technology and its impact on the surrounding bone, also requires histological evaluation of the bone‐implant interface. Histology allows proper assessment of not only the relative extent of bone‐to‐implant contact and bone marrow but also the possible presence of inflammation fibrous tissue, or any other aberrant events.

As already discussed above, small animal platforms are considered convenient and cost‐effective screening tools to evaluate the potential of novel implant technology to enhance osseointegration. The increased amount of information produced from a single experiment in a small animal platform, using the proposed two‐piece implant design, may assist in a more qualified decision‐making process, on whether it is relevant to proceed to further assessment using a large animal platform. Furthermore, the increased volume of information generated in a single animal experiment either in a small or large animal platform, using the proposed two‐ and three‐piece implants, respectively, likely decreases the number of animals otherwise needed for collecting the same information with standard one‐piece implants. Thus, the use of these novel two‐ and three‐piece implants likely contributes to the reduction/refinement elements of the 3R principle (Russell & Burch, 1959), a legal requirement in current EU legislation (Directive 2010/63; http://ec.europa.eu/environment/chemicals/lab_animals/legislation_en.htm).

## AUTHOR CONTRIBUTIONS

**Andreas Stavropoulos**, **Benjamin Bellon**, **Benjamin Pipenger**, and **Ole Z. Andersen**: Conceptualization, presentation, drafting of the manuscript.

## CONFLICT OF INTEREST STATEMENT

B. B., O. Z. A., and B. P. are employees of Institut Straumann, the industrial partner of ITI.

## ETHICS STATEMENT

The clinical images are from different preclinical studies, approved by the relevant animal ethics authorities.

## Data Availability

Data sharing is not applicable to this article as no data sets were generated or analyzed during the current study.
